# A New Speech-in-Noise Test for Measuring Informational Masking in Speech Perception Among Elderly Listeners

**DOI:** 10.7759/cureus.7356

**Published:** 2020-03-21

**Authors:** Marzieh Amiri, Farnoush Jarollahi, Shohreh Jalaie, Seyyed Jalal Sameni

**Affiliations:** 1 Department of Audiology, School of Rehabilitation Sciences, Iran University of Medical Sciences, Tehran, IRN; 2 Physiotherapy, School of Rehabilitation Sciences, Tehran University of Medical Sciences, Tehran, IRN

**Keywords:** informational masking, perceptual masking, elderly, speech recognition

## Abstract

Introduction

Elderly listeners have reported concerns about speech perception in noisy environments. This partly occurs because of their increased informational masking (IM). This study aimed to develop a Persian coordinate response measure (CRM) corpus and a novel speech-in-noise test for measuring IM.

Material and methods

A cross-sectional validation study was conducted in two parts. Part one was the determination of the validity and reliability of the Persian CRM corpus. Part two consisted of measuring the IM at five signal-to-noise ratio (SNR; -6,-3, 0, +3, and +6) in two conditions: one with the target and masker speaker of the same sex and one with the target and masker speaker of different sexes. In each condition, the IM measurements were performed at a 45° separation angle of target and maskers and as a co-location of the speakers. A group of young listeners aged 20 to 40 years and a group of elderly listeners aged 60 to 75 years were recruited (50 study participants in part one and 47 in part two). The study was conducted from July 2018 to March 2019 at the Iran University Medical Sciences audiology clinic. Content validity ratio, content validity index, impact score, Spearman's test, and Mann-Whitney's test were used for statistical analysis.

Results

The Persian CRM corpus showed acceptable validity and reliability in each group (p < 0.001). The results suggested that in both azimuth locations and at SNRs of 0, -3, and -6, the IM amount in the elderly group was significantly higher (p < 0.003) than in the young group at conditions of target and masker speakers of opposite-sex. However, in cases where both target and masker speakers were of the same sex, a significant difference was observed at an SNR of 0 in angular separation and SNRs of +3 and 0 at co-located situations (p < 0.001).

Conclusion

A validated Persian CRM corpus has been collected for use in IM measurement studies. Overall, the IM of elderly listeners was higher than younger listeners in low-cue situations such as lower SNR. Therefore, a novel speech-in-noise test for measuring IM was validated to use in speech perception studies in the elderly population.

## Introduction

The process by which the threshold of hearing for one sound is raised by the presence of another sound is called masking. In our everyday environment, as we listen to meaningful signals around us, we typically encounter masking in the form of unwanted background noise. For example, during an oral presentation, ambient sounds from air-conditioning systems, the murmur of side conversations, and/or audible sounds from outside the presentation room such as traffic or weather often interfere with (i.e., mask) the instructor. Some studies have reported that the masking occurs more intensely in situations with competing-speech maskers as opposed to steady-state maskers. In situations with competing-speech maskers, two types of masking occur: energetic masking (EM) and informational masking (IM) [1-5]. EM is a consequence of temporal and spectral overlap of the target and competing signals [2]. IM is the cognitive processing interference between target and distractors, also referred to as non-EM or perceptual masking [3-5]. Therefore, in the context of speech, IM is deﬁned as excess masking that cannot be explained by spectral interference between the target and masker. While EM arises because of frequency selectivity limitations at the peripheral level, IM reﬂects processing capacity limitations at a more central level [6]. Some investigations have shown that, depending on their linguistic information, competing signals have different masking effects on target speech perception. For example, competing speech affects the perception of target speech more than other unintelligible distractors such as time-reversed speech. Moreover, native competing signals impair the processing of target signals more significantly than non-native speech [5]. In this case, increased competition occurs at the higher levels of the auditory processing system.

Two main factors affect the amount of IM: stimulus uncertainty and similarity between the target and masker [7]. In these situations, perceptual segregation may be reduced, preventing the listener from performing auditory scene analysis. To understand how susceptibility to IM changes across the lifespan, we must examine the performances of elderly people and young adults alike. Elderly listeners may have a reduced ability for selective listening, resulting in poorer listening in environments that contain competing speech. Additionally, auditory spatial processing (ASP) plays an important role in auditory scene analysis and perception of speech in complicated noisy situations [8]. In fact, younger participants learn to form auditory streams and analyze the auditory scene to segregate between the target and competing signals [9-11]. It seems that angular separation between the target and competing signals is the most important cue regarding the release of IM [10]. ASP is poorer in elderly people compared with young adults [12,13], and age-related changes in ASP and binaural processing are not entirely related to peripheral hearing loss [14,15]. Factors beyond hearing loss, such as changes in temporal processing or general slow processing speed, may limit the use of spatial cues among normal-hearing elderly listeners [14-16]. On the other hand, investigations have shown that older people require higher signal-to-noise ratios (SNRs) to track speech in noisy situations. It means that they need a higher strength of the signal carrying information to that of unwanted interference. These studies suggest that the poorer performance of older adults in noisy environments is probably caused by a modality-specific decline in cognitive processing such as the reduced ability to use acoustic and phonetic cues [17].

Currently, speech-in-noise (SIN) tests are important components of the battery of audiology tests and hearing research. SIN tests are designed to mimic real-life circumstances and also can provide valuable information about a person's hearing ability. Audiologists often use SIN tests in terms of parameters such as target age, measure, procedure, speech material, and noise, among others [18]. Various tests are available to estimate speech perception in the presence of noise, but none of them directly measure the amount of IM. Although there is a body of research on IM, there is no test specifically designed to measure IM [5,6,12,13,16,17,19]. Methods to differentiate between EM and IM have scarcely been described. Given IM’s important role in noisy situations, developing a new test to measure it would be useful. On the other hand, such tests can be useful tools to evaluate the effectiveness of rehabilitation programs focused on speech perception improvement before and after treatment. They can also help clinicians gain better insight into the nature of elderly speech pathology.

As mentioned above, there are some limited ways to differentiate between EM and IM. By using speech as the competing distractors, both EM and IM can be created. But using unintelligible speech distractors such as time-reversed speech create high EM and little IM. Therefore, the amount of IM can be calculated by comparing speech perception between these situations [12,13]. Different studies investigating IM have used a host of speech materials such as consonant-vowel and monosyllabic or disyllabic words and sentences. Among those, coordinate response measure (CRM) sentences appear to offer a good option to differentiate between EM and IM [13,19]. These sentences are relatively context-free, and by using them as the target and competing signals, a high semantic and syntactic similarity is observed, which is an important factor in introducing IM [19]. Since no appropriate Persian materials are available for the evaluation of IM, the first objective of this study was to develop the Persian CRM sentences and to determine their content and face validity. After preparing the phrases, the reliability of the Persian CRM corpus in two groups of young and elderly normal-hearing listeners was determined. The second objective of this survey was to develop an IM measurement (IMM) test by using the Persian CRM sentences. To determine the diagnostic validity of the IMM test, the results of two groups of young and elderly listeners with normal pure-tone audiograms were compared.

## Materials and methods

Participants

This survey was a cross-sectional validation study consisting of two main parts. In part one, the Persian version of a CRM corpus was prepared, and its validity and reliability were determined in a group of 50 people including 25 young (11 males and 14 females) and 25 elderly listeners (12 males and 13 females) The young group was aged 20 to 40 years (mean age: 24 years; standard deviation [SD]: 5 years) and the elderly group was aged 60 to 75 years (mean age: 66 years; SD: 2.96). In part two, the IM amount was assessed in a group with a similar age range consisting of 24 young listeners (mean age: 26 years; SD: 6 years) and 23 elderly listeners (mean age: 66 years; SD: 5 years). In both parts, the younger group consisted of college students from the Iran University of Medical Science (IUMS), whereas the elderly group consisted of patients referred to the IUMS audiology clinic. Elderly group educational distribution was as follows: 50 percent had diploma, 40 percent had BS degree, and 10 percent had MS degree. Both parts were conducted at the IUMS audiology clinic. Testing took place in a double-walled anechoic chamber.

The inclusion criteria for all participants were auditory thresholds ≤ 25 dB within the 250-4,000 Hz frequency range, with no frequency worse than 40 dB to ensure a normal pure-tone audiogram, and a word recognition score of ≥96% in quiet [20]. Pure tone and speech audiometry were assessed by AC40 Audiometer (Interacoustic, Middelfart, Denmark). Additional criteria included the absence of cognitive problems using the Persian version of the Mini-Mental Status Exam questionnaire (cutoff point set as a score of 23), having a diploma or higher academic degree, right-handedness (using the Edinburgh handedness inventory), speaking Farsi and being monolingual, the absence of reported concerns about SIN perception difficulties, and the lack of pathology of the external and middle ear (otoscopy and tympanometry [AZ26 clinical impedance audiometer, Interacoustic] was used to evaluate the middle ear function [type An was chosen as a normal middle ear function]) [21,22]. Exclusion criteria included previously diagnosed hearing loss people, current hearing aid users (within the last six months), and the unwillingness to participate in every step of the intended research and/or not meeting the other inclusion criteria.

Study procedures

Part One: Preparing and Determining the Validity and Reliability of the Persian Version of CRM Corpus

The same rigid structure with “Ready [call-sign] go to [color] [number] now” format was used in CRM phrases. In the English version of the CRM, eight call-signs, four colors, and eight numbers from one to nine were used to form the phrases. These sentences were expressed by different male and female talkers [13,19].

In the first step, the sentences were translated into Persian. Eight Persian monosyllabic numbers from one to nine, four monosyllabic Persian colors, and ten disyllabic Persian male names were selected (Table 1).

**Table 1:** The Persian CRM corpus used in this study CRM, coordinate response measure

Ready "call-sign"	Go to "color"	"Number" now
Ali	Zard (yellow)	1
Hossein	Sabz (green)	2
Arad	Sorkh (red)	3
Artin	Beige (Beige)	5
Mahan		6
Mahdi		7
Reza		8
Sobhan		9
Yasin		
Taha		

To determine the quantitative amount of content validity, the content validity ratio (CVR) and content validity index (CVI) were calculated. First, the prepared phrases associated with the prepared questions for the evaluation of content validity were emailed to five audiologists, and they were asked to score each question as “essential,” “not essential but useful,” or “not essential.” Second, to determine the CVI, the prepared phrases and questions were mailed to 20 experts including audiologists, speech-language pathologists, and linguistics, who are academic members of the IUMS Rehabilitation Faculty, Tehran University of Medical Sciences, and Shahid Beheshti University of Medical Sciences. These experts were asked to score the content validity on a Likert scale (Table 2).

**Table 2:** The questionnaire on content and face validity for the Persian version of CRM corpus CRM, coordinate response measure

Content validity		Totally appropriate	Appropriate	Not appropriate	Suggestions
	Sentence translation into Persian				
	Choosing the call-signs				
	Choosing the numbers				
	Choosing the colors				
Face validity	Sentences’ spoken rate				
	Sentence understandability				
	Sentence clarity				

In the following step, four talkers (two men and two women) were asked to record the Persian CRM corpus. A total of 240 sentences were created for each talker (10×3×8). All of the sentences were recorded in a studio in accordance with the main criteria mentioned for recording the English version including sampling rate of 44.1 kHz, three seconds for recording each sentence, removing the initial and final silence gaps in the recorded sentences, scaling all the words in CRM such that they occur simultaneously (i.e., coordinate sentences), and filtering the sentences using a band-pass filter within the range of 80 to 8,000 Hz [19,23]. The recorded sentences were then given to the same experts mentioned above. To determine the face validity, the experts were asked to fill a questionnaire (Table 2). Finally, the impact scores of the recorded sentences were evaluated.

To evaluate the reliability of the Persian CRM phrases, the correct recognition scores of CRM sentences in silence were evaluated between the two study groups in two different one-hour sessions at two-week intervals. The test was conducted at the most comfortable hearing level. Each person listened to 120 sentences in each session. Each talker, color, number, and call-sign was repeated 30, 40, 15, and 12 times, respectively. Each sentence was given a correct score when both its number and color were identified correctly (total score). The talker, color, number, and call-sign error rates were measured separately in the first session. Stimuli were played from a sound card (Sound Blaster X-Fi, Creative Labs, Milpitas, CA, USA) and sent to the appropriate loudspeaker (Pejvak Ava Corporation, Tehran, Iran) in front of the participant at 0-degree azimuth.

Part Two: Developing and Determining the Validity of the IMM Test

One of the best ways to evaluate IM is to compare the speech recognition score between two competing signals: intelligible and unintelligible [6,16,24]. In this study, the CRM percent-correct score was measured under two scenarios.

CRM was used as competing signals (intelligible maskers). CRM percent-correct score was measured in the presence of two different CRM sentences. The competing CRM sentences are different in call-sign, color, and number from the target signal. The listeners were trained to respond to the target signal, which has the call-sign “Ali” and ignore the other sentences with different call-signs. The participant had to recognize the color and number of the target sentence.

Time-reversed speech was used as competing signals (unintelligible maskers). CRM competing signals were manipulated using a time-reversed technique to eliminate their intelligibility. Moreover, the long-term spectra of the stimuli remained fixed; therefore, the EM was preserved but the IM was reduced. To eliminate the intelligibility, the spoken sentence had to be broken down into more than 40-millisecond (ms) successive segments, as intelligibility is known to decline when successive segment duration is prolonged [25]. In our case, the segment duration was 300 ms. MATLAB R2018 software (MathWorks, Inc., Natick, MA, USA) was used to construct the time-reversed CRM sentences.

To assess IM amount, the target CRM sentence was always presented from a loudspeaker in the 0-degree azimuth, and two competing CRM sentences were presented simultaneously from loudspeakers at ±45 degrees and 0-degree azimuth (once collocated and once spatially separated from the target). The competing sentences were played at a fixed sound pressure level of 60 dB, and the target sentence intensity was adjusted to produce the specified SNRs (±6, ±3, and 0 dB). In all cases, sex was once considered similar for the competing and target signals and once different. Therefore, the amount of IM was measured under a total of 20 conditions: five SNRs, two spatial conditions, and two talker sexes. The formula for calculating IM in each condition was as follows:

CRM percent-correct score in the intelligible competing signals - CRM percent-correct score in unintelligible competing signals = IM score (amount)

To determine the diagnostic validity of the IMM test, two groups of young and elderly participants were recruited. The diagnostic validity of a test refers to its ability to differentiate between persons with and without a specified condition or disorder [26]. Participants attended one 1.5-hour session. At the beginning of each session, five sentences were chosen from the Persian CRM sentences and presented to familiarize the participants with the conditions. These sentences were different from those that were used in the test sessions. The investigator instructed participants to recall the color and number of the target sentence with call-sign “Ali” and ignore the other competing signals. Ten sentences were presented in each of the test conditions. In each session, anytime a participant needed a break, the session stopped for 10 minutes before continuing.

Statistical analysis

IBM SPSS Statistics for Windows, Version 20.0 (IBM Corp., Armonk, NY, USA) was used for statistical data analysis, and the significance level for all tests was set at 0.05. In the descriptive analysis, central tendency and dispersion indices (mean and SD) were used. CVR and CVI were used to determine content validity. The impact score was used to measure face validity. The Kolmogorov-Smirnov test revealed that the data do not have a normal distribution; therefore, nonparametric tests were used. To determine the reliability of the Persian CRM corpus, the Spearman test was employed. In the second part, the Mann-Whitney test was conducted to compare the results between the two groups.

Sample size

To determine the sample size for the first part of this study, we referred to a study by Terwee et al. that suggested that at least 50 participants must be enrolled to evaluate reliability [27]. In total, 50 participants aged 20 to 75 years with normal pure-tone audiograms were recruited in the first part of this study.

The following formula was used to determine diagnostic validity in the study’s second part:

$n = \frac{(Z_{1-\alpha/2}+Z_{1-\beta})^{2}(S_{1}^{2}+S_{2}^{2})}{(\mu _{1}-\mu _{2})}$

S1: Standard deviation of the studied variable in the first group (young group)

S2: Standard deviation of the studied variable in the second group (elderly group)

µ1: Mean of the studied variable in the first group

µ2: Mean of the studied variable in the second group

α=0.05

β=80%

In this formula, the studied variable is the amount of IM. We found no previously published study that used the same test like ours, and therefore we performed a pilot study. The sample size calculated indicated 20 participants in each group. A total of 24 young and 23 elderly listeners were recruited.

Ethics

The Medical Ethics Committee of IUMS approved the study protocol. Written informed consent was obtained from all participants. The purpose of the research was explained to all participants. Data confidentiality was ensured. Participants were given a choice to withdraw their participation at any time and were ensured that all conducted tests had no foreseeable side effects and were free of charge.

## Results

Part one: preparing and determining the validity of the Persian version of CRM corpus

As mentioned above, the quantitative values of content and face validity of the Persian CRM sentences were measured. The CVR of the prepared questions of the questionnaire was 1. CVR values range from −1 (i.e., perfect disagreement) to +1 (i.e., perfect agreement), and CVR values above 0 indicating that more than half of panel members agree that an item is essential. Therefore, a CVR value of 1 indicates that questions were prepared appropriately. Individual item CVI (I-CVI) and overall scale CVI (S-CVI) were then calculated. Except for the color beige, the I-CVI and S-CVR of all keywords were 1; therefore, the color beige was deleted. The impact score of all recorded sentences was 4. As the accepted value of the impact score was 1.5, it was concluded that the recorded speech materials had appropriate face validity.

**Figure 1:**
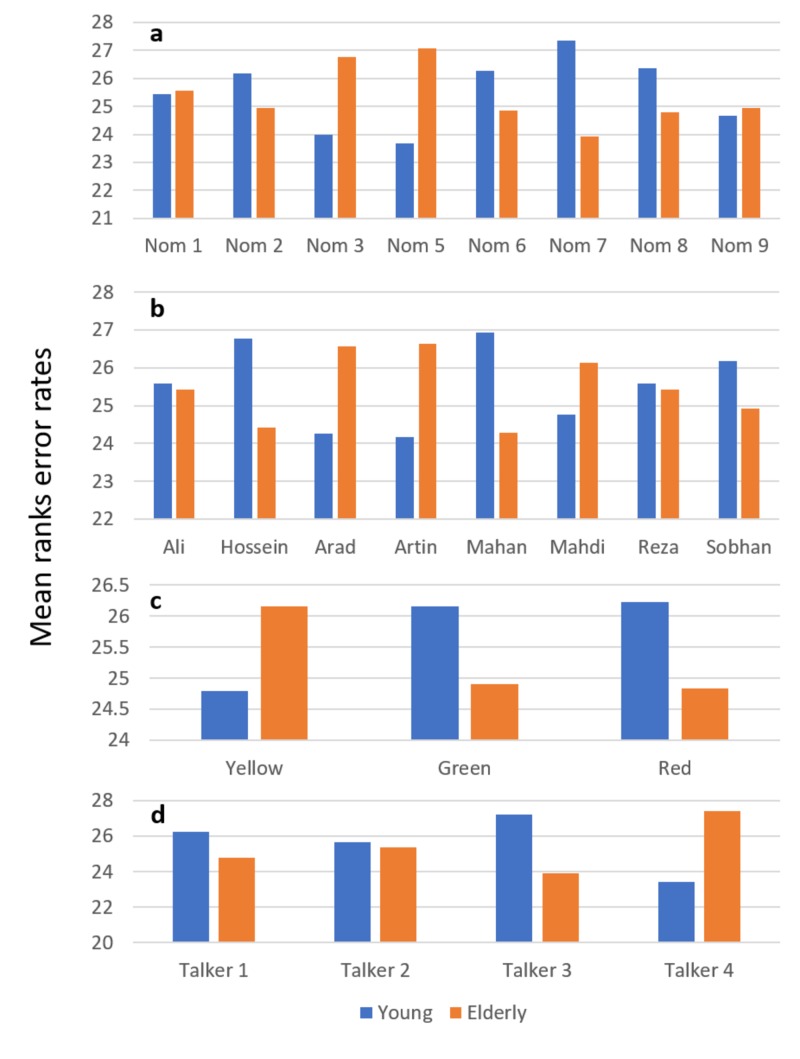
Mean ranks error rates for numbers (a), call-signs (b), colors (c), and talkers (d). Talker error rates are measured as error rates of sentences in which the color-name combination was misidentified. Nom, number

A total of 50 individuals were recruited in this part of the study (25 young and 25 elderly). These people participated in a two-session assessment. In the first session, error rates for call-signs, colors, and numbers per talker were calculated separately. Figure 1 depicts the distribution of mean ranks error rates by keyword and across talkers. The Mann-Whitney test was used to compare error rates between the two groups. There were no significant differences between the two groups for all keywords and talkers (p > 0.05). The Friedman test was performed to indicate differences within each group for all keywords and talkers. For example, in the elderly group, talker four had the highest error rate, but there was no significant difference between the mean rank error rates of each talker in this group (p = 0.08) (Figure 1). There was no significant difference between the other keywords in each group.

To determine reliability, a retest was administrated after a two-week interval. Spearman’s correlation coefficient was calculated in each age group to determine the test-retest reliability. There was a significant correlation between the test-retest results in each group (p < 0.05). Spearman’s correlation coefficients were 0.786 (p < 0.001) and 0.898 (p < 0.001) in the young and elderly groups, respectively.

Part two: developing and determining the validity of the IMM test

Two groups of people participated in this part of the study (24 young and 23 elderly). The Mann-Whitney test was conducted to compare the IM amount and CRM correct recognition score under two maskers (intelligible and unintelligible noise). The results of comparing all 60 conditions between the two groups can be found in Table 3. At most of the positive SNRs (+3, +6), no significant differences were observed between the two study groups. In both azimuth locations and at SNRs of 0, -3, and -6, the IM amount in the elderly group was significantly higher (p < 0.03) than that of the younger group under conditions where the sex of the target signal differed from that of the maskers. This means that elderly people had a poorer performance compared with younger participants under these conditions. However, when both the target and masker were of the same sex, only a significant difference was observed at an SNR of 0 in angular separation and at SNRs of +3 and 0 at co-located situations (p = 0.00).

In the intelligible competing situations, two kinds of masking occurred, but when unintelligible maskers were used, the EM was reduced and, consequently, the IM amount was increased. When comparing the CRM correct recognition scores in the intelligible competing conditions, there was a significant difference between both groups (at SNRs of 0, -3, and -6; p < 0.006). But the CRM percent-correct score in the unintelligible maskers (EM) slightly differed depending on the sex of the masker. In the case of maskers with different sex, the performance of elderly participants did not differ from the younger ones in the spatially separated conditions (p = 0.209). However, in the co-located condition, there was a significant difference between the two groups (p=0.027). As can be seen in Table 3, in the case of talkers of the same sex, the performance of the elderly group was poorer than that of younger adults in both spatial conditions (p = 0.00).

**Table 3:** Comparison of all 60 conditions evaluated in part two between the two study groups. Values are presented as mean ± standard deviation (n = 47). SNR, signal-to-noise ratio; IMDS45, informational masking amount for different-sex talkers at 45-degree azimuth separation; IMDS0, informational masking amount for different-sex talkers in the co-located condition; IMSS45, informational masking amount for same-sex talkers at 45-degree azimuth separation; IMSS0, informational masking amount for same-sex talkers in the co-located condition; CRMDS45, CRM recognition score for different-sex talkers at 45-degree azimuth separation; CRMSD0, CRM recognition score for different-sex talkers in the co-located condition; CRMSS45, CRM recognition score for same-sex talkers at 45-degree azimuth separation; CRMSS0, CRM recognition score for same-sex talkers in the co-located condition; TRSDS45, time-reversed speech recognition score for different-sex talkers at 45-degree azimuth separation; TRSDS0, time-reversed speech recognition score for different-sex talkers in the co-located condition; TRSSS45, time-reversed speech recognition score for same-sex talkers at 45-degree azimuth separation; TRSSS0, time-reversed speech recognition score for same-sex talkers in the co-located condition

Condition	SNR: +6			SNR: +3			SNR: 0				-3				-6		
	Young	Elderly	p-Value	Young	Elderly	p-Value	Young	Elderly	p-Value	Young		Elderly	p-Value	Young		Elderly	p-Value
IMDS45	0.00±0.00	0.00±0.00	1.000	0.00±0.00	0.43±2.08	0.307	0.00±0.00	5.21±7.90	0.001	1.25±3.37		6.08±6.56	0.002	4.16±5.03		13.04±12.58	0.004
IMDS0	0.00±0.00	0.00±0.00	1.000	0.00±0.00	0.43±2.08	0.307	1.25±4.48	6.08±7.22	0.004	5.41±7.21		11.36±10.99	0.008	6.66±9.16		15.21±13.09	0.030
IMSS45	0.00±0.00	0.00±0.00	1.000	1.66±4.81	3.40±5.72	0.156	10.83±10.59	23.47±14.33	0.003	20.00±13.83		19.13±16.21	0.039	2.41±14.58		19.13±15.92	0.445
IMSS0	0.41±2.04	2.60±7.51	0.262	3.33±9.16	10.43±11.66	0.006	18.33±11.67	31.30±13.24	0.001	14.58±17.18		22.60±17.34	0.000	18.33±17.36		13.47±15.84	0.312
CRMDS45	100.00±0.00	100.00±0.00	1.000	100.00±0.00	99.56±2.08	0.307	100.00±0.00	94.34±9.45	0.001	97.91±4.14		90.43±10.21	0.536	90.00±12.15		71.73±26.73	0.006
CRMDS0	100.00±0.00	100.00±0.00	1.000	100.00±0.00	99.13±4.17	0.307	97.91±6.58	90.86±9.00	0.001	92.91±8.66		80.43±21.42	0.015	81.25±11.90		58.26±25.34	0.001
CRMSS45	100.00±0.00	100.00±0.00	1.000	97.50±6.75	96.52±5.72	0.299	83.75±13.12	71.73±15.85	0.011	73.75±14.98		60.86±20.43	0.000	59.58±15.17		30.86±25.39	0.000
CRMSS0	99.58±2.04	97.39±7.51	0.262	95.00±11.79	84.78±16.20	0.007	69.58±15.45	44.34±19.96	0.000	64.41±18.41		23.47±24.60	0.000	47.91±19.33		10.00±14.45	0.000
TRSDS45	100.00±0.00	100.00±0.00	1.000	100.00±0.00	100.00±0.00	1.000	100.00±0.00	99.56±2.08	0.307	98.75±3.37		99.52±8.31	0.655	95.83±5.83		85.21±24.65	0.209
TRSDS0	100.00±0.00	100.00±0.00	1.000	100.00±0.00	99.56±2.08	0.307	99.16±2.82	99.95±6.34	0.180	98.33±3.80		91.30±13.91	0.158	87.91±11.02		73.04±95.12	0.027
TRSSS45	100.00±0.00	100.00±0.00	1.000	99.16±2.82	100.00±0.00	0.162	94.58±8.32	94.78±6.65	0.817	93.75±6.46		79.56±14.60	0.050	80.00±10.63		54.34±27.60	0.000
TRSSS0	100.00±0.00	100.00±0.00	1.000	98.33±3.80	95.21±8.45	0.205	88.33±9.16	75.65±14.71	0.002	80.00±10.63		46.08±27.42	0.000	67.08±13.34		23.47±24.42	0.000

## Discussion

Part one: preparing and determining the validity of the Persian version of CRM corpus

In this study, the Persian version of the CRM was prepared to assess speech perception among two groups of young and elderly listeners. The I-CVI and S-CVR of all keywords were 1. The impact score of all recorded sentences was 4. Therefore, the content and face validity of the Persian CRM corpus were acceptable. All keywords had the same effect on intelligibility, and there were no significant differences between the four talkers. Evaluation of the test-retest reliability revealed that the Persian CRM corpus had acceptable reliability in the young and elderly groups.

As reported by Wilson et al., using SIN tests is very important in the regular auditory test battery [28]. The available SIN tests do not evaluate the perceptual processes used to perceive speech under more complicated listening conditions involved in IM. This study was conducted to develop a new SIN test to evaluate the IM amount in two groups of young and elderly listeners. Since the first step in any SIN test development is stimuli selection, the investigator should be careful in that selection as it can directly affect the nature of the test. The performance of the test can be affected by the type of speech material used [27]. The nature of the CRM corpus suggests that these speech materials are a good choice in assessing speech intelligibility in multichannel communication environments [19,23]. In preparing the Persian CRM corpus, we tried to follow the criteria of the original version. The 10 most frequently disyllabic masculine names were collected as call-signs along with 3 monosyllabic Persian colors and 8 monosyllabic Persian numbers. Therefore, a total of 240 sentences were prepared for each talker. Because of the need for precise control of stimulus onset and the large number of talkers required, digital recordings of CRM phrases are preferable to live talkers and, therefore, we used digital recordings [13,19,23]. The methods of collecting and recording the Persian CRM sentences were the same as those used by Bolia et al. [23]. The content and face validity of the Persian CRM corpus were acceptable, making it an applicable choice in research and clinical studies. There were no significant differences between the four talkers. As mentioned in the Results section, although in the elderly group, talker 4 had the highest error rate (Figure 1), there was no significant difference between the mean rank error rates of each talker in this group. Accordingly, a very suitable Persian CRM corpus consisted of 960 sentences available for speech perception investigations. The evaluation of the test-retest reliability revealed that the Persian CRM corpus had acceptable reliability in the young and elderly groups. To the best of our knowledge, there is currently no other published data on the examination of the validity and reliability of the CRM corpus in silence.

Part two: developing and determining the validity of the IMM test

The main method of isolating the IM component of speech in multi-talker situations is to use two or three kinds of background noises: intelligible and unintelligible maskers [13,19]. However, no specific test is available to evaluate the IM. Therefore, this part of our study used the Persian CRM corpus to develop a new IM evaluation test.

At the most positive SNRs, older adults can overcome the background noise and perceive the target message. By using the SIN test in a group of normal-hearing elderly listeners, Shojaei et al. found that speech intelligibility was increased by about 14% by increasing the SNR from 0 to +10 dB. They concluded that SNRs had a critical role in speech perception ability in the elderly [29]. Some researchers also found that the difficulty of older people in SIN perception is more obvious at more adverse SNRs [16]. Hence, in our study, SNR is an important factor to overcome masking in the elderly group, which have normal hearing according to the results of their audiograms.

In both azimuth locations and at SNRs of 0, -3, and -6, the IM amount in the elderly group was significantly higher than that of the younger group under different-sex conditions for the target and maskers. Elderly participants had a poorer performance compared with younger participants in these situations, which is in agreement with some previous studies. For example, Rajan and Cainer found that in participants older than 60 years, speech perception thresholds of sentences in the presence of noise were increased. The authors concluded this was a modality-specific decline in cognitive processing such as a decrease in using acoustic and phonetic cues to segregate speech from noise [17]. This is also consistent with the work of previous researchers who suggested that IM occurred more intensely in the elderly when the target and maskers were highly similar and aligned. They also suggested that these differences between younger and older participants were especially higher at more adverse SNRs [1,16], which is also consistent with our findings.

In same-sex situations for both the target and maskers, the IM amount was significantly different only at an SNR of 0. This may seem confusing at first because according to the theories of age-related changes, we expect age differences to grow larger as the similarity between the target and masker increases [30]. Hence, we expect that for same-sex talkers, there must be a significant difference between the two groups at all adverse SNRs. Interestingly, a similar result was reported recently by researchers who found that speech recognition differences between older and younger normal-hearing adults were significant for opposite-sex stimuli but not when the target and masker were of the same sex [30]. This finding is also supported by Helfer and Freyman [1]. They found that although young listeners experience minimal problems when the masker is of the opposite sex than the target, older adults experience considerable difficulty in such situations [1]. On the other hand, in the case of same-sex talkers, the younger group also had difficulties in understanding the speech. By using CRM phrases in a group of 21- to 55-year-old listeners, Brungart found that the best performance was achieved for different-sex maskers, and performance declined when same-sex maskers were used [13]. This means that in our study, in the same-sex maskers and at adverse SNRs, both younger and older listeners experience a higher level of IM.

By comparing the CRM correct recognition score in the situations in which both EM and IM occurred (i.e., competing conditions where the maskers were intelligible), in all conditions, there was a significant difference between both groups (at SNRs of 0, -3, and -6). This finding agrees with Helfer and Freyman, who reported that all older participants had poorer performances than younger ones at varying degrees under every noisy condition [1]. The elderly group in our study had poorer performance under these competing situations for both EM and IM. However, the results were slightly different when we compared the CRM percent-correct score in the situations only affected by EM (i.e., when the distractors were unintelligible). In the case of different-sex maskers, the performance of older adults did not differ from that of the younger ones in the spatially separated conditions, but in the co-located condition there was a significant difference between the two groups. Therefore, older adults can overcome the EM by using some cues such as spatial separation and noticing the opposite-sex of talkers. Nevertheless, in the case of same-sex talkers, the CRM percent-correct score under both spatial situations differed between the two groups. Hence, in same-sex situations, the spatial cue did not help older adults overcome EM.

Our results indicate that older adults generally experience difficulty when the masker is presented, regardless of whether the masker produces IM. This finding is in agreement with previous studies on speech perception in noise [20,28]. Heidari et al. compared SIN test results between two groups of young and elderly normal-hearing listeners and reported that speech processing in older adults is deteriorated compared with that of younger ones [20].

Our study had three important limitations. Most elderly people may suffer from varying degrees of presbycusis; therefore, there is limited research on the elderly listeners with normal pure-tone audiograms. Finding the normal pure-tone audiogram of elderly listeners was also a limitation of this study. Also, we did not use any tests such as otoacoustic emission and auditory brainstem response to recognize the cochlear hidden hearing loss (i.e. damage in the cochlea despite normal hearing).

## Conclusions

A Persian CRM corpus containing recorded multi-talker sentences has been collected to support behavioral and psychoacoustic studies in speech perception. This study reveals that the speech perception ability of normal pure-tone audiogram elderly listeners is considerably reduced in the presence of meaningful background noise. Moreover, decreasing SNRs significantly reduces the perceptual abilities of elderly individuals in all conditions. SNR is critical for elderly listeners to mitigate masking for proper speech perception in a noisy listening environment. ASP and binaural processing might play important roles in speech perception in elderly listeners. Audiologists may need clinical tools such as the IMM test to evaluate speech perception in the presence of different background noises and angular azimuths in elderly normal, pure-tone audiogram listeners. On the other hand, these kinds of tests can be useful tools to evaluate the effectiveness of the rehabilitation programs focused on speech perception improvements before and after treatment and can give clinicians better insight into the nature of elderly speech perception deficits.
